# A survey on large language models in biology and chemistry

**DOI:** 10.1038/s12276-025-01583-1

**Published:** 2026-04-08

**Authors:** Islambek Ashyrmamatov, Su Ji Gwak, Su-Young Jin, Ikhyeong Jun, Umit V. Ucak, Jay-Yoon Lee, Juyong Lee

**Affiliations:** 1 Research Institute of Pharmaceutical Science, College of Pharmacy, Seoul National University, Seoul, Republic of Korea; 2 Graduate School of Data Science, Seoul National University, Seoul, Republic of Korea; 3 Department of Molecular Medicine and Biopharmaceutical Sciences, Graduate School of Convergence Science and Technology and College of Pharmacy, Seoul National University, Seoul, Republic of Korea; 4 Arontier co., Seoul, Republic of Korea

**Keywords:** Computational models, Machine learning

## Abstract

Artificial intelligence (AI) is reshaping biomedical research by providing scalable computational frameworks suited to the complexity of biological systems. Central to this revolution are bio/chemical language models, including large language models, which are reconceptualizing molecular structures as a form of ‘language’ amenable to advanced computational techniques. Here we critically examine the role of these models in biology and chemistry, tracing their evolution from molecular representation to molecular generation and optimization. This review covers key molecular representation strategies for both biological macromolecules and small organic compounds—ranging from protein and nucleotide sequences to single-cell data, string-based chemical formats, graph-based encodings and three-dimensional point clouds—highlighting their respective advantages and inherent limitations in AI applications. The discussion further explores core model architectures, such as bidirectional encoder representations from transformers-like encoders, generative pretrained transformer-like decoders and encoder–decoder transformers, alongside their sophisticated pretraining strategies such as self-supervised learning, multitask learning and retrieval-augmented generation. Key biomedical applications, spanning protein structure and function prediction, de novo protein design, genomic analysis, molecular property prediction, de novo molecular design, reaction prediction and retrosynthesis, are explored through representative studies and emerging trends. Finally, the review considers the emerging landscape of agentic and interactive AI systems, showcasing briefly their potential to automate and accelerate scientific discovery while addressing critical technical, ethical and regulatory considerations that will shape the future trajectory of AI in biomedicine.

## Language models drive innovation in biomedical discovery

Large language models (LLMs) are artificial intelligence models that understand and generate human language. Scientists want to use LLMs to better understand complex scientific data, but there are challenges because scientific data differ from human language. Researchers reviewed how LLMs are being adapted for tasks in chemistry and biology by training them using large datasets, such as protein sequences and chemical structures. This study highlights the importance of designing effective representations that LLMs can understand, which is crucial for their success in scientific applications. The results reveal that LLMs can predict protein structures and design new molecules, potentially revolutionizing drug discovery and related areas. The researchers conclude that while progress has been made, more work is needed to align natural-language LLMs to fully address scientific needs.

This summary was initially drafted using artificial intelligence, then revised and fact-checked by the author.

## Introduction

Large language models (LLMs), built on deep neural architectures and trained on massive text corpora, have achieved state-of-the-art performance in language understanding, generation and reasoning. Although originally developed for natural language, their core modeling principles are broadly transferable to symbolic scientific data. This has spurred growing interest in adapting LLMs to scientific domains, particularly in chemistry and biology^1,2^.

Scientific knowledge and understanding critically depend on the construction of formal representations that encode the structure and behavior of physical and biological systems. These representations are designed for fidelity in capturing domain-specific properties but rarely align with the distributional and syntactic patterns of language models. Thus, various attempts have been suggested for better alignment between LLMs and scientific representations^3,4^.

What enables LLMs to perform so effectively is not an understanding of individual tokens but their ability to model the statistical structure that governs token composition. In scientific domains, a model’s ability to infer properties depends on how well the input representation encodes underlying structure. Thus, representational design is not peripheral but fundamental for developing scientific LLMs. It determines what models can learn, generalize and, ultimately, discover. In addition, it is well known that the scales of model architecture and training data are critical in accuracy and emergent behaviors of LLMs^5^. Therefore, the success of scientific LLMs rests on both the scale and the architecture of the models and how effectively the representation translates a domain structure into a learnable entity.

Recent progress in using LLMs in biology and chemistry has been accelerated by the growth of curated, domain-specific datasets. Molecular and protein databases, along with scientific literature, now support diverse training strategies, from self-supervised objectives to multimodal integration. However, much of this development remains fragmented, and systematic comparisons across chemical and biological domains are still limited.

**Fig. 1:**
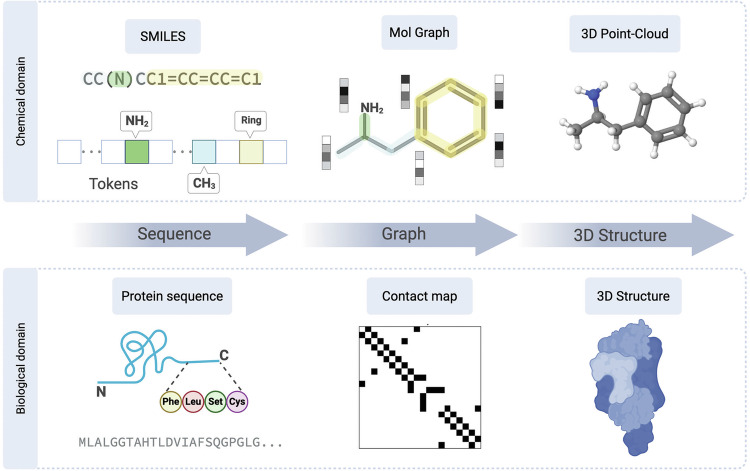
A comparative overview of molecular representation modalities. Molecular information can be encoded at multiple levels of abstraction. In the chemical domain (top), a molecule is represented as a one-dimensional (1D) SMILES string, a 2D topological graph or a 3D spatial point cloud. Analogously, in the biological domain (bottom), a protein is represented by its 1D amino acid sequence, a 2D contact map of residue proximities or its folded 3D structure. The choice of representation dictates the complexity and type of information available to a language model.

Here, we examine how LLMs are being adapted to the unique demands of chemical and biological topics. We focus on how representations, architectures and training regimes influence model performance across domains and tasks. The foundational challenge lies in converting complex, multidimensional molecular information into formats that language models can process (Fig. 1). Our goal is to clarify what has been achieved, what remains challenging and how these models will better serve scientific understanding.

## Biological language models

The unprecedented success of LLMs has opened a new paradigm in data analysis. In the field of biology, the utilization of various biological data such as protein sequences^6^, structures^7^, nucleotides^8^ and species taxonomy^9^ has been considered. The application of transformer architectures to biological problems has led to substantial breakthroughs, with AlphaFold2 (AF2)^10^ and RoseTTAFold^11^ emerging as landmark models in protein structure prediction. In parallel, ongoing research is being conducted to describe biological complexity more accurately within the models (Table 1).

**Table 1:** Categorization of biological language, structure and multimodal models.

Category	Modality		Name	Architecture
Modality specific	ProteinQ	Protein sequence	ProtBERT, MSA Transformer, ESMFold	Transformer (encoder only)
			ProtGPT2	Transformer (decoder only)
			ProGen, ProGen2	Transformer (encoder–decoder)
			ProteinMPNN^**^	Message-passing neural network^*^
			ProtMamba	Mamba^*^
		Protein structure**	AlphaFold2, RoseTTAFold	Biology-specific modules^*^
			RFdiffusion	Diffusion model (RF architecture)^*^
	Nucleotide	DNA sequence	DeepSite	CNN, LSTM
			DNABERT, GROVER, DNABERT2	Transformer (encoder only)
			MegaDNA	Transformer (decoder only)
			HyenaDNA	Hyena^*^
			Caduceus	Mamba^*^
		RNA sequence	GenSLM	Transformer
	Single cell	scRNA sequence	scBERT, Geneformer	Transformer (encoder only)
			scGPT	Transformer (decoder only)
			scELMo, GenePT	Pretrained LLM
			CancerGPT	Fine-tuned LLM
Multimodality	Protein sequence, protein structure, protein function		ESM3	Transformer (encoder only)
	DNA sequence, RNA sequence		Evo	Hyena^*^ (StripedHyena)
	Protein, nucleic acid, ligand		AlphaFold3	Biology-specific modules^*^
	Image, text		BiomedGPT	Transformer (encoder–decoder)
	Protein, molecule, text		BioMedGPT-10B	Transformer (decoder only)
	Molecule, protein, nucleic acid, cell, disease, text		Tx-LLM	Transformer (decoder only)

Biological language, structure and multimodal models can be categorized on the basis of data modality, architecture and biological specificity.

Single-modality data were applied to the modality-specific model, while several modalities of data were used in the multimodal model. The modality includes protein, nucleotide and single cell, and it includes biological data such as a single sequence and 3D structure and general data including text and image. ^*^Indicates models being customized to reflect biological characteristics using existing architecture. ^**^The modality is classified on the basis of the characteristics of the output.

### Protein language models

The sequential nature of protein has enabled the application of language modeling techniques from natural language processing. Early models such as ProtBERT^12^, MSA Transformer^13^ and ProtTrans^14^ leveraged core techniques from the deep language models while exploring variations in both input formats, for example, single sequences, multiple sequence alignments (MSAs) and architectures, for example, unidirectional and BERT-style bidirectional encoders. ESMFold^2^ achieves AF2-level accuracy in protein structure prediction without relying on MSAs, capturing contextual dependencies solely through language modeling. The scaling of model parameters and faster structure prediction highlight the potential of language models when trained on large-scale biological data. ProtMamba^15^ also showed that protein language modeling is feasible without MSAs. The model adopts a state space architecture based on Mamba^16^ instead of on attention to handle long-range sequences.

Protein design aims to generate proteins with completely new functions and structures, and generative models can play a key role in the process. ProGen^17^ enables controlled protein sequence generation by incorporating conditioning tags into an autoregressive transformer architecture. ProGen2^18^ and ProtGPT2^19^ further improve upon previous models by leveraging more complex conditioning tags to generate sequences that satisfy both structural and functional constraints. Recently, diffusion architectures, developed for image generation from text prompts, have been adapted for protein structure generation. RFdiffusion^20^ incorporates spatial constraints through SE(3) equivariance, enabling more efficient and physically consistent sampling of protein structures. Such structural modeling has facilitated scaffolding tasks, and tools including ProteinMPNN^21^ and Foldseek^22^ have accelerated advances in protein design.

### Protein structure models

Protein structure models predict the tertiary structures of proteins from their primary amino acid sequences. Traditional techniques such as X-ray crystallography, NMR spectroscopy and cryo-electron microscopy have been used to elucidate protein structures. However, these experimental methods are often constrained by high costs, time requirements and technical limitations, resulting in a considerably slower accumulation of structural data compared to the rapidly expanding number of known protein sequences^23^. This sequence–structure data imbalance (for example, between UniProtKB^24^ and the Protein Data Bank^7^) underscores the need for computational prediction approaches to complement experimental efforts.

AlphaFold (AF)^25^ and AF2^10^ have demonstrated outstanding performance in the field of protein structure prediction, as indicated by their success in Critical Assessment of Protein Structure Prediction 13 and 14, respectively. AF2 consists of two primary modules: the Evoformer and the structure module. Unlike AF, which employs a ResNet-based convolutional neural network (CNN), AF2 introduces an attention-based Evoformer, enabling efficient processing of MSAs and pairwise residue interactions. The Evoformer can be interpreted as a biology-specific transformer, where MSAs are treated as sequences in natural language, capturing evolutionary patterns across homologous proteins. This approach has been more fully realized in protein language models, which are designed to replace MSAs by implicitly modeling evolutionary information. The structure module allows for end-to-end learning from primary sequence to three-dimensional (3D) structural reconstruction, achieving near experimental accuracy.

Several platforms have been developed to extend the applicability and accessibility of protein structure models. ColabFold^26^ leverages a metagenomic sequence database (ColabFoldDB) to enhance the diversity and quality of MSAs, and it is implemented to run on web-based graphics processing unit resources through Google Colaboratory. This approach improves accessibility to high-accuracy protein structure prediction while effectively reducing computational resource burdens. Phyre2.2^27^ is an upgraded web portal for protein structure and function prediction that maintains a user-friendly interface while integrating AF-predicted structures as new templates. It enables large-scale structural analysis by utilizing a broader range of structural templates beyond those available in the Protein Data Bank. Furthermore, it supports domain-level optimization and batch-mode prediction, thereby serving as a computational alternative that complements experimental studies.

### Nucleotide language models

Unlike natural language, DNA does not possess an inherent concept of ‘words’, and its composition is limited to just four nucleotides—adenine (A), thymine (T), guanine (G) and cytosine (C)—as opposed to protein sequences, which are composed of approximately 20 amino acids. This limited alphabet reduces the overall information density, making the development of effective DNA language models more challenging.

Earlier approaches, such as DeepSite^28^, utilized CNNs and recurrent neural networks for modeling DNA sequences. However, CNNs often struggle with capturing long-range dependencies, and recurrent neural networks suffer from computational inefficiency and scalability issues. To address these limitations, DNABERT^29^ adopted a masked language modeling (MLM) based on bidirectional encoder representations from transformers (BERT) using *k*-mer tokenization (that is, n-gram in computer science), enabling more effective sequence representation. Subsequent models, including GROVER^30^ and DNABERT2^31^, leveraged byte pair encoding (BPE)^32^—tokenization used by the SentencePiece^33^ framework—to flexibly define token units. This helped to reduce sequence information loss and improved computational efficiency. As a result, transformer-based models have been successfully applied to tasks such as identifying promoters and transcription factor binding sites directly from DNA sequences. Caduceus^34^ uses character-level (base-pair) tokenization, which ensures robustness to minor sequence variations. Furthermore, by modeling DNA sequences bidirectionally and incorporating reverse complement equivariance, Caduceus demonstrates superior performance on tasks such as regulatory site prediction and long-range single nucleotide polymorphism effect inference. Recently, research has been performed beyond MLM toward generative approaches, such as MegaDNA^35^, which is a transformer-based DNA sequence generation model.

GenSLM^36^ is an RNA language model capable of mutation effect prediction by capturing the differences between original and mutated RNA sequences and predicting their functional effects. The model uses a codon-level vocabulary, which avoids frame-shift issues, for tokenizing RNA sequences. The study addresses input lengths that exceed the standard maximum capacity of the standard transformer. This limitation has been identified as a fundamental architectural bottleneck in early foundation models designed for nucleotide sequence analysis. Evo^37^, HyenaDNA^38^ and Caduceus^34^ have adopted specialized architectures, such as Hyena^39^ and Mamba, to support long-sequence modeling.

### Single-cell language models

With the accumulation of high-dimensional gene expression data, single-cell language models have emerged as a new frontier in biology. While proteins and nucleotides are naturally sequential, single-cell gene expression data are not universally sequential. Therefore, a method of ranking genes on the basis of their expression levels has been proposed. Genes within a cell are treated as words in a sentence, and Transformer-based models are applied to capture their underlying dependencies, as in other biological language modeling tasks.

Recent advances in single-cell representation learning have surpassed traditional marker gene-based approaches in capturing cellular heterogeneity^40^. scBERT^41^ addresses this limitation by leveraging full gene expression profiles, achieving strong performance in cell type annotation. Geneformer^42^ handles the nonsequential nature of gene expression data by ordering genes on the basis of count statistics, also showing effectiveness in classification tasks. Building on this, scGPT^43^ takes gene embeddings as input tokens and outputs a cell embedding, jointly learning representations at both levels. It achieves state-of-the-art results across tasks such as cell type classification, perturbation prediction, batch correction and multiomics integration. These findings emphasize the value of large-scale single-cell datasets (for example, the Human Cell Atlas^44^ and CellMarker^45^) and the potential of embedding models to capture cellular complexity.

At the same time, approaches have been proposed to leverage general-purpose LLMs for directly incorporating prior biological knowledge, going beyond gene sequence modeling alone. For example, despite being trained on common human languages, Generative Pretrained Transformer (GPT)-4 has shown the ability to perform automatic cell type annotation on the basis of text prompts describing gene expression levels^46^. Accordingly, GenePT^47^ and scELMo^48^ have constructed gene- and cell-level embeddings by applying text embedding application programming interfaces (APIs) from a corpus of biomedical literature, including the National Center for Biotechnology Information database. It has been reported to outperform some biological data-driven models such as Geneformer^42^. In addition, CancerGPT^49^, a GPT-3^50^ model fine-tuned on corpora of text, predicts drug response pairs within rare tissue types by aligning textual representations with cellular information. Developing disease-specific models with refined cell embeddings may further advance precision medicine.

### Biomolecule representations

Biological macromolecules such as proteins and nucleic acids can be represented through diverse modalities to support machine learning applications. Sequence-based representations use amino acid or nucleotide strings and serve as the foundation for protein and genomic language models such as ESM^2^, ProtBERT^12^ and DNABERT^29,31^. Structural representations capture spatial information using atomic coordinates, contact maps or distance matrices, which are leveraged in structure models such as AF and ESMFold. Graph-based approaches abstract biomolecules into nodes and edges, enabling the use of geometric deep learning models such as SE(3) Transformer^51^. Functional representations include gene ontology terms, protein family annotations and subcellular localization, enriching models with biological context. At the cellular level, omics data such as scRNA-seq are encoded as high-dimensional expression vectors.

### Tokenization strategies

Unlike human language, biological sequences lack clear semantic boundaries and often have ambiguity and nested structures. Therefore, tokenization methods have evolved from traditional machine learning techniques, including *k*-mer approaches^52^, to biomolecule-specialized strategies such as structure- and codon-based tokenization^53^, which are critical for accurate and detailed biomolecular modeling. In protein and nucleotide models, *k*-mer tokenization (for example, 3-mer and 6-mer) is used to capture local biochemical context, as seen in DNABERT and ProtBERT. Some models use BPE or unigram models trained on large corpora of sequences, such as DNABERT2, ESM and ProGen. Codon-based or codon-preserving tokenization are also adopted to avoid frame-shift artifacts in nucleotide modeling. scBERT employs the gene2vec approach to generate gene embeddings, which facilitates the application of the BERT architecture to single-cell RNA sequencing data. These customized strategies ensure efficient representation of biological syntax and semantics in pretrained language models.

### Application of BLMs in biomedicine

#### Integrative modeling for molecular cell biology

AF2 demonstrated the strength of artificial intelligence in protein structure prediction and has since inspired a wide range of follow-up studies. Models such as AF3^54^, RoseTTAFoldNA^55^ and RoseTTAFold All-Atom^56^ extend their focus beyond proteins to include other biologically relevant molecules such as RNA, DNA and ligands. In particular, all-atom structure prediction introduces computational challenges in accurately reconstructing 3D coordinates. This reflects a growing recognition that structural accuracy is essential for understanding biomolecular function not only in proteins but also in RNA, where structure plays a critical role in regulatory activity^57^. Concurrently, LLM-based methods have begun to incorporate structural information, moving beyond sequence modeling. ESM3^58^ jointly embeds sequence, structure and function, marking a transition toward multimodal representation. Specialized models such as ESM-DBP^59^ have also been developed to predict DNA-binding proteins, adopting hybrid approaches that leverage both sequence and structure features. In the context of unified modeling in BLMs, foundation models aim to learn comprehensive cellular representations by integrating diverse biological modalities. These include epigenetic marks, spatial transcriptomics, protein expression data and perturbation signatures, which can be explored to gain a deeper understanding into cellular function^60^. The integration signals a broader shift from modality-specific models toward unified representations that more reasonably reflect the inherent complexity of biological systems.

#### Multimodal foundation models

Multimodal LLMs offer a framework for aligning heterogeneous data types, such as clinical notes, protein sequences and molecular structures.

BiomedGPT^61^ aligns natural language with biomedical modalities, particularly visual representations, to enable cross-modal reasoning for vision-language tasks. It focuses on applications such as diagnosis, summarization and clinical decision support through flexible query answering. However, such models still exhibit limitations in reasoning across complex clinical scenarios, including the interpretation of radiological images and the resolution of textual conflicts. MediConfusion^62^ provides a diagnostic benchmark that systematically evaluates failure modes of multimodal medical LLMs.

**Fig. 2:**
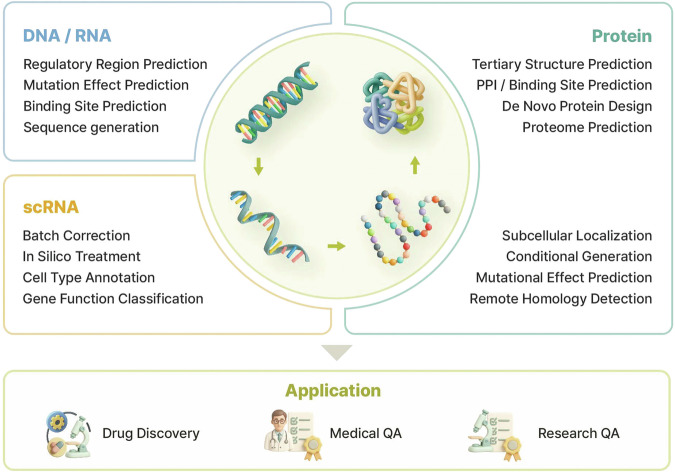
Downstream tasks of BLMs. The diagram organizes downstream tasks of BLMs according to data modalities (DNA/RNA, protein and single-cell RNA) and illustrates their practical applications in drug discovery, medical QA and scientific research.

Tx-LLM^63^ leverages the advantages of large-scale pretraining on diverse biological datasets. Specifically, it is trained on sequence-level information encompassing RNA, DNA, protein sequences as well as Simplified Molecular Input Line Entry System (SMILES). This comprehensive approach enables positive transfer performance in end-to-end drug discovery tasks, outperforming models that do not incorporate biological sequence data. Similarly, BioMedGPT-10B^64^ contributes to drug discovery by specializing in protein and molecule question and answering (QA), having been trained on cell sequences as well as protein and molecule structures. These advancements highlight the potential of LLMs to serve as unified multimodal platforms in biomedicine (Fig. 2).

## Chemical language models

Chemical Language Models (CLMs) have been suggested to learn the structure–activity relationship of small molecules from large-scale chemical data using various sequential representations of molecules, for example, SMILES^65^.

### Model types

**Fig. 3:**
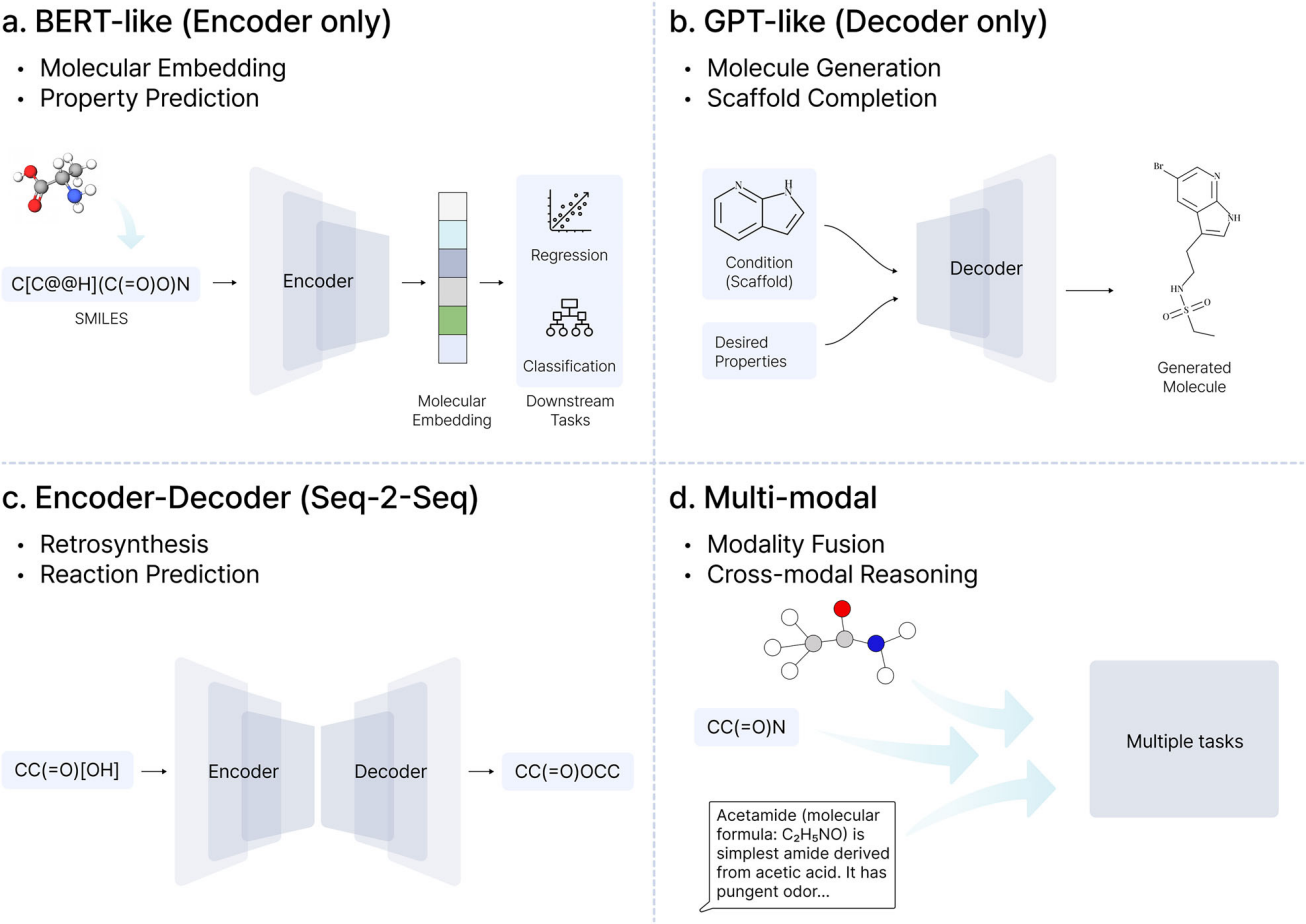
Representative architectures of CLMs. **a**, Encoder-only models are trained to learn well-representing molecular embeddings, supporting subsequent downstream tasks. **b**, Decoder-only models generate molecules autoregressively, enabling de novo design or conditional generation. **c**, Encoder–decoder models handle structured sequence-to-sequence mappings, which is effective for retrosynthesis and reaction prediction. **d**, Multimodal models combine SMILES, graphs and various types of data to enable integrated molecular reasoning across modalities.

Similar to protein language models, most CLMs leverage Transformer architectures^66^, akin to those in natural language processing, to understand, generate and manipulate chemical structures and reactions. These models are broadly categorized on the basis of their architectural design, each optimized for distinct tasks within cheminformatics and drug discovery. The primary model types include encoder-only (BERT-like) models, decoder-only (GPT-like) models and encoder–decoder architectures, as well as emerging multimodal LLMs that integrate diverse data formats (Fig. 3). These architectural choices dictate how the models process molecular representations and perform tasks ranging from property prediction to de novo molecular design and retrosynthesis.

#### Chemical encoders

Encoder-only transformer models, primarily inspired by BERT, are designed to extract contextual representations of molecules and are well suited for property prediction and molecular understanding. ChemBERTa^67^ adapts the RoBERTa^68^ framework with MLM and multitask regression, where auxiliary property prediction tasks are defined using molecular features computed by RDKit^69^. Mol-BERT^70^ applies MLM to learn chemically informed token-level dependencies and is fine-tuned for tasks such as property classification and activity prediction. MoLFormer^71^ extends this approach using linear attention and rotary embeddings, yielding compact representations useful for downstream regression and classification tasks, although it is limited to relatively small molecules. Further encoder variants refine token representations or integrate structural priors. MolRoPE-BERT^72^ enhances positional encoding, while MFBERT^73^, SELFormer^74^ and semi-RoBERTa^75^ introduce architectural modifications for greater chemical expressiveness. Graph-enhanced encoders such as GROVER^76^ incorporate topological features directly, bridging the gap between sequence and graph representations.

#### Chemical decoders

Decoder-only transformer models, following the GPT architecture, are optimized for autoregressive generation and have become essential in de novo molecular design. MolGPT^77^ prioritizes causality to learn token-wise dependencies and ultimately generates novel molecules. It supports conditional generation strategies to bias outputs toward specific chemical properties. GP-MoLFormer^78^ is a decoder-only adaptation of MoLFormer-XL^71^ and is optimized for tasks such as unconstrained molecule generation, scaffold completion and conditional property optimization. Other GPT-based chemical models include SMILES-GPT^79^ and iupacGPT^80^, which are both adapted from GPT-2^81^ for molecular and nomenclature sequence generation. cMolGPT^82^ extends this framework for controllable generation under property or scaffold constraints. Taiga^83^ combines GPT modeling with reinforcement learning to guide molecule synthesis toward multiobjective goals.

#### Encoder–decoder architectures

Encoder–decoder transformer models are designed for sequence-to-sequence tasks, making them particularly effective for applications such as retrosynthesis, reaction prediction and cross-domain molecular translation. Text+ChemT5^84^ uses a shared encoder–decoder T5 backbone to support dual-modality tasks across chemical and natural language domains, including text-to-molecule generation and vice versa. SELFIES-TED^85^, built upon a BART-style encoder–decoder structure, is tailored for chemically constrained generation tasks. It consistently performs well across molecular prediction and generative benchmarks, showing strong generalizability.

Beyond these, Chemformer^86^ and BARTSmiles^87^ adopt the BART architecture for generative and discriminative molecular tasks. MOLGEN^88^ introduces self-feedback during pretraining to better align model output with chemically realistic constraints. Models such as Molecular Transformer^1^, Retrosynthesis Transformer^89^ and SCROP^90^ focus on forward and backward reaction prediction, employing techniques such as snapshot learning, syntax correction and beam search to enhance accuracy and syntactic validity. Hybrid approaches have also emerged: GO-PRO^91^ integrates context-free grammars, RetroTRAE^92^ tracks atom-level transformations via fragment tokenization and GCT^93^ augments the Transformer with a conditional variational autoencoder for latent sampling. Prompt-driven models such as RetroSynth-Diversity^94^ and the Disconnection-Aware Transformer^95^ further refine retrosynthesis by guiding outputs on the basis of fragmentation strategies or disconnection heuristics.

#### Multimodal LLMs

Chemical information is inherently multimodal, encompassing textual descriptions, molecular graphs, two-dimensional (2D) depictions, 3D coordinates and higher-dimensional properties, such as polarizability. Standard CLMs, designed to handle only text format, cannot fully capture heterogeneous information. To address this, recent CLMs integrate LLMs with structural encoders to enable cross-modal reasoning. Mol-LLaMA^96^ incorporates graph representations into a language model, improving tasks such as functional group identification and retrosynthesis. GIT-Mol^97^ processes graphs, images and text through separate encoders and then fuses their modality-specific tokens via a shared representation layer. Contrastive objectives are used to align modalities, and a joint prediction head enables multitask learning across modalities. LLM-MPP^98^ similarly aligned SMILES, 2D graphs and text descriptions through cross-attention and contrastive learning to enable coherent molecular representation. Vision-language models such as PRESTO^99^ and ChemVLM^100^ jointly encode molecular depictions and associated texts to support synthesis planning and reaction condition inference. nach0^101^ treats SMILES, images and text as aligned modalities within a shared representation space for multimodal reasoning. Collectively, these approaches reflect the expanding range of design strategies aimed at achieving effective modality fusion within CLMs.

### Pretraining and fine-tuning strategies

#### SSL

Self-supervised learning (SSL) is a powerful subset of unsupervised learning where labels are automatically generated from the input data itself. This approach is commonly used for pretraining models on large, unlabeled datasets, which is crucial for ensuring the generalizability of the learned representations. In this regard, MLM is a widely adopted pretraining task for encoder-based language models. In this approach, a certain percentage of tokens (for example, 15%) in the input sequence are randomly masked, and the model is trained to predict these masked tokens on the basis of the surrounding context^102^. This forces the model to learn deep contextual representations and implicitly understand the underlying chemical syntax and molecular structures, which can then be transferred to various downstream tasks. Denoising objectives are another form of SSL where the model is trained to reconstruct the original, clean input from a corrupted or ‘noisy’ version^103,104^.

#### Multitask learning

Multitask learning is a powerful paradigm that utilizes shared information across multiple related learning tasks to improve generalization and overall performance. By training a single model on several tasks simultaneously, it is compelled to learn common patterns and representations that are beneficial across all tasks^105^. This approach can be conceptualized as machines mimicking human learning, where knowledge acquired from one task can effectively benefit and improve performance on other related tasks^106^. Multitask learning is particularly advantageous in molecular prediction tasks, as it helps alleviate data sparsity issues by allowing models to draw strength from diverse but related datasets, leading to improved accuracy. Models such as Text+ChemT5 exemplify this by being multidomain, multitask language models that concurrently handle both chemical and natural language. They achieve this by sharing weights across these distinct domains and tasks, fostering a unified understanding. Similarly, nach0-pc^107^ is a multitask language model specifically designed for 3D molecular structures, demonstrating its capability to process complex point cloud data effectively within a multitask framework

#### Retrieval-augmented generation

Retrieval-augmented generation enhances language models by integrating a latent retriever that dynamically accesses external documents during pretraining, fine-tuning and inference^108^. In chemistry, this modular architecture improves performance on tasks such as molecule design, retrosynthesis and reaction prediction, with reported gains of up to 17.4% over standard inference^109^. It also mitigates hallucinations by grounding predictions in up-to-date domain-specific data^110^. However, conventional retrieval-augmented generation often neglects structural dependencies among retrieved documents. Models such as ATLANTIC^111^ address this by building heterogeneous document graphs and using frozen graph neural networks (GNNs) for context encoding, thus improving retrieval quality while preserving computational efficiency.

#### Supervised fine-tuning

Supervised fine-tuning adapts pretrained CLMs to specific tasks using labeled datasets. It aligns model outputs with experimental annotations through continued gradient-based optimization, supporting applications such as property prediction, reaction classification and synthesis planning. While full-model fine-tuning often yields strong performance, it can be computationally intensive and prone to overfitting in low-resource settings. To mitigate these issues, several parameter-efficient alternatives have emerged, including adapter tuning, prefix tuning, prompt tuning and LoRA (Low-Rank Adaptation)^112^, which constrain the number of trainable parameters while maintaining task adaptability. These methods offer scalable alternatives that retain the benefits of large-scale pretraining. Regardless of strategy, fine-tuning success critically depends on data quality, which remains central to achieving reliable and interpretable outcomes.

### Molecular representations

Just as natural language processing relies on effective text tokenization and embedding, CLMs depend on robust and informative molecular representations. This section explores the primary representation schemes, detailing their principles, advantages and the challenges they pose for artificial intelligence systems.

#### String-based representations

SMILES^65^ encodes molecular structures as linear ASCII strings, originally developed for efficient data storage. Its brevity, machine-readability and invertibility have made it widely adopted in cheminformatics and compatible with language models. However, SMILES presents several limitations when used with LLMs. A key issue is nonuniqueness: a single molecule can have multiple valid SMILES representations, which complicates training and reduces model generalization. Canonicalization reduces this ambiguity but often encourages overfitting to syntactic patterns rather than learning underlying chemical rules. SMILES also lacks explicit stereochemistry and 3D spatial information, and many datasets omit annotations for chiral centers and geometric isomers, limiting its utility in structure-sensitive tasks. To address these shortcomings, several extensions to the SMILES grammar have been proposed, including DeepSMILES^113^, SELFIES^3^ and Atom-in-SMILES^4^, with particular attention to improving validity, interpretability and compatibility with machine learning systems.

#### Graph-based representations

Graph-based inputs capture connectivity and topological constraints that are absent in SMILES, offering richer structural context. Models such as GROVER^76^ and MG-BERT^114^ incorporate GNN-derived embeddings or graph-attention mechanisms to bridge this gap. Despite their promise, graph-based hybrid CLMs face challenges in tokenization, alignment with sequence models and limited standardized pipelines. Ongoing efforts focus on improving graph serialization, integrating positional encodings for graphs and combining GNNs with pretrained transformers in parameter-efficient ways.

#### 3D point cloud representations

Recent advances have extended CLMs beyond linear and 2D representations by incorporating explicit 3D molecular structure, particularly via point cloud-based methodologies. These models exploit geometric deep learning to capture spatial features that are critical to tasks such as molecular property prediction and drug design. Notably, models range from specialized 3D-aware transformers such as Uni-Mol^115^ to multimodal architectures such as nach0-pc^107^ and 3D-MolT5^116^ that combine molecular point cloud encoders with language models to learn from atomic spatial arrangements via joint training on multitask 3D datasets. These approaches represent a shift toward spatially grounded CLMs with an improved capacity to model complex molecular geometry and interactions.

### Tokenization strategies

Tokenization in CLMs refers to the transformation of molecular strings into discrete, model-readable units. Unlike general natural language processing, token boundaries in chemistry must respect atomic symbols, charges and bonding syntax. For instance, character-level tokenization is ill-suited for SMILES, as it often produces tokens that represent unphysical or chemically meaningless characters. Canonical schemes such as BPE^32^, while effective in natural language processing, tend to merge chemically unrelated substrings under frequency pressure. To address this, domain-specific approaches such as Atom Pair Encoding^117^ or token frequency regularization have been proposed. Atom-in-SMILES^4^ tokenization embeds local topological context—such as neighboring atoms or ring membership—into tokens, improving resolution without altering syntax. This yields more balanced token distributions and enhances optimization performance in low-data regimes.

### Applications of CLMs in biomedicine

CLMs are being increasingly used across biomedicinal research, particularly in drug discovery. These models predict molecular properties such as solubility, bioavailability and toxicity directly from string representations, allowing rapid screening of candidate compounds and reducing dependence on experimental assays. CLMs also support de novo molecule generation^118^. Autoregressive and diffusion-based models can design novel compounds with optimized activity, selectivity or synthetic accessibility. In biomedicine, such tools are applied to generate inhibitors, antibiotics and central nervous system-targeted molecules tailored to therapeutic needs.

In chemical synthesis, CLMs predict reaction outcomes and assist in retrosynthetic planning. Sequence-to-sequence models trained on reaction corpora such as USPTO^119^ suggest plausible routes for synthesizing drug-like molecules, improving efficiency and creativity in medicinal chemistry^120^. CLMs further aid early toxicity assessment by learning structural patterns linked to adverse effects. When trained on toxicology datasets, they support preclinical risk evaluation more effectively than rule-based methods^121^. These applications reflect how CLMs contribute to faster and more informed decision-making in biomedicinal pipelines.

## Datasets for bio/chemical language models and benchmarks

The effectiveness of language models in biological and chemical domains is closely tied to the diversity, structure and scale of training data. Numerous datasets have been curated to support tasks such as molecular property prediction, reaction modeling, clinical text understanding and biomedical question answering.

Chemical structure databases such as ZINC^122^, PubChem^123^ and ChEMBL^124^ provide millions of small molecules in SMILES format, supporting the learning of chemical syntax and structure–activity relationships. For modeling reactivity and synthesis, datasets such as USPTO^119^, Reaxys^125^, QM9^126^ and QMugs^127^ offer extensive reaction and quantum property annotations. To evaluate predictive performance across physical and bioactivity tasks, benchmarks such as MoleculeNet^128^ (ESOL, FreeSolv, Lipophilicity, Tox21, SIDER, BBBP and HIV) are widely adopted.

On the biomedical side, large corpora such as PubMed^129^, PubMed Central^130^ and clinical datasets including MIMIC-III^131^, eICU^132^ and i2b2^133^ enable models to learn domain-specific language and clinical reasoning patterns. Complementary benchmarks—such as MedQA^134^, PubMedQA^135^, BioASQ^136^ and MultiMedQA^137^—serve for evaluating medical question answering and multihop inference capabilities. For therapeutic science and knowledge extraction, specialized datasets such as Therapeutics Data Commons^138^, DisGeNET^139^, DrugBank^140^, PHARMGKB^141^ and STRING^142^ offer structured annotations across gene–disease, drug–target and protein–interaction networks.

## The use of LLMs in biology and chemistry

Prompt engineering has emerged as the most accessible way to adapt ChatGPT and other LLMs to scientific problems without additional training. This method relies on carefully crafted textual prompts to direct model outputs, with applications ranging from SMILES translation to molecular property prediction. Techniques such as zero-shot, few-shot and chain-of-thought prompting^143^ have demonstrated utility across diverse chemical tasks. Liu et al.^144^ showed that performance can be surprisingly strong for tasks such as retrosynthesis planning and reaction classification. However, prompt sensitivity, limited domain knowledge and inconsistent outputs remain major limitations^145,146^. Despite these drawbacks, prompt engineering has facilitated rapid, low-resource adaptation of LLMs to domain-specific tasks.

For greater task specificity, fine-tuning offers a more robust route. By continuing pretraining on chemical corpora—including scientific literature and curated datasets—ChatGPT-like models can internalize domain language and logic. Studies have demonstrated improved performance in chemical property regression, reaction prediction and literature-based knowledge extraction^147,148^. Domain-specific fine-tuning, even on modest datasets, has shown effectiveness in areas such as inorganic chemistry or thermoelectrics^149,150^. Nevertheless, outcomes are highly sensitive to dataset quality and task design^151,152^.

At the frontier, ChatGPT has been combined with agentic systems that allow LLMs to use tools, yielding multistep workflows and autonomous decision-making. Approaches such as ReAct^153^ orchestrate LLM reasoning with external tool use. Coscientist^154^, ChemCrow^155^ and ChatMOF^156^ exemplify such frameworks, integrating web search, retrosynthesis tools and laboratory protocols into interactive agents. In automation contexts, platforms such as Chemputer^157^ and Organa^158^ have demonstrated LLM-guided synthesis planning and laboratory execution. These systems promise scalable scientific automation but remain dependent on deterministic toolchains and human oversight.

## Conclusion

The convergence of large-scale data and advanced computation has established a new paradigm in the molecular sciences, reconceptualizing both biological and chemical systems as structured languages amenable to deep learning. At the core of this transformation is the shared challenge of representation: translating complex, multidimensional molecular information—from protein sequences and single-cell expression profiles to SMILES strings—into formats that learning architectures can effectively process. As detailed in this review, this has led to advanced models for predicting protein structure, interpreting genomic regulation and performing de novo molecular design and synthesis planning.

This development reflects a broader shift toward unified, multimodal frameworks that integrate diverse data types to build more comprehensive and robust foundation models. As these architectures mature, future progress will depend critically on aligning model capabilities with fundamental prior biological and chemical knowledge during the learning process. The development of standardized benchmarks for rigorous model evaluation and improvement of model interpretability to build trust and guide experimental validation are also essential.

Looking ahead, the development of bio/chemical LLMs is heading toward more interactive and agentic systems capable of assisting with hypothesis generation and experimental design. By successfully addressing the aforementioned challenges, these bio/chemical language models are poised to become foundational platforms for creating more predictive, generative and reliable tools, which will substantially accelerate the design–build–test–learn cycle in molecular science.
